# The clinical and cost effectiveness of group art therapy for people with non-psychotic mental health disorders: a systematic review and cost-effectiveness analysis

**DOI:** 10.1186/s12888-015-0528-4

**Published:** 2015-07-07

**Authors:** Lesley Uttley, Matt Stevenson, Alison Scope, Andrew Rawdin, Anthea Sutton

**Affiliations:** School of Health and Related Research (ScHARR), University of Sheffield, Regent Court, 30 Regent Street, Sheffield, S1 4DA UK

**Keywords:** Art therapy, Mental health disorders, Psychological therapy, Systematic review, Health technology assessment, Cost-effectiveness

## Abstract

**Background:**

The majority of mental health problems are non-psychotic (e.g., depression, anxiety, and phobias). For some people, art therapy may be a more acceptable alternative form of psychological therapy than standard forms of treatment, such as talking therapies. This study was part of a health technology assessment commissioned by the National Institute for Health Research, UK and aimed to systematically appraise the clinical and cost-effective evidence for art therapy for people with non-psychotic mental health disorders.

**Methods:**

Comprehensive literature searches for studies examining art therapy in populations with non-psychotic mental health disorders were performed in May 2013. A quantitative systematic review of clinical effectiveness and a systematic review of studies evaluating the cost-effectiveness of group art therapy were conducted.

**Results:**

Eleven randomised controlled trials were included (533 patients). Meta-analysis was not possible due to clinical heterogeneity and insufficient comparable data on outcome measures across studies. The control groups varied between studies but included: no treatment/wait-list, attention placebo controls and psychological therapy comparators. Art therapy was associated with significant positive changes relative to the control group in mental health symptoms in 7 of the 11 studies. A de novo model was constructed and populated with data identified from the clinical review. Scenario analyses were conducted allowing comparisons of group art therapy with wait-list control and group art therapy with group verbal therapy. Group art-therapy appeared cost-effective compared with wait-list control with high certainty although generalisability to the target population was unclear; group verbal therapy appeared more cost-effective than art therapy but there was considerable uncertainty and a sizeable probability that art therapy was more cost effective.

**Conclusions:**

From the limited available evidence art therapy was associated with positive effects compared with control in a number of studies in patients with different clinical profiles. The included trials were generally of poor quality and are therefore likely to be at high risk of bias. Art therapy appeared to be cost-effective versus wait-list but further studies are needed to confirm this finding in the target population. There was insufficient evidence to make an informed comparison of the cost-effectiveness of group art therapy with group verbal therapy.

**Trial registration:**

HTA project no. 12/27/16; PROSPERO registration no. CRD42013003957.

## Background

Mental ill health is recognised as the largest cause of disability in the United Kingdom [1]. The UK Department of Health have prioritised making mental health services more effective and accessible in response to evidence that such services are not meeting the needs of some groups of people [2, 3]. The majority of mental health problems are non-psychotic (e.g., depression, anxiety, and phobias). For some people with these conditions, art therapy may be an acceptable alternative form of psychological therapy than more standard forms of treatment, such as talking therapies [4]. For example, for those who find it difficult to express themselves in verbal language alone as required by more standard forms of treatment for mental health problems, arts therapies can provide an alternative means of expression to help service users understand, make sense of, and cope with their distress. There is some published evidence to support the claim that art therapy is effective in treating a variety of symptoms and disorders in patients of different ages [5, 6]. However, to date a full systematic review of the clinical and cost-effectiveness of art therapy for non-psychotic mental disorders had not been undertaken. This project aimed to systematically review the current clinical and cost-effectiveness evidence for art therapy for people with non-psychotic mental disorders. In addition, a *de novo* cost-effectiveness analysis would be undertaken if the systematic review did not identify suitable studies.

Art therapy is a specific branch of treatment under the umbrella term “arts therapies” used by the Health Care Professions Council (HCPC) which includes drama therapy and music therapy. For the purpose of this review these other forms of arts therapies, which do not centre on the creation of a sustainable, physical piece of visual art, are excluded. Despite art therapy being an established and practised form of psychological therapy for decades, only more recently have researchers in the field of art therapy addressed the need to integrate art therapy into a model of evidence-based practise. Therefore, an abundance of literature exists consisting of single case studies or theoretical concepts in art therapy [7]. This study was part of a health technology assessment commissioned by the National Institute for Health Research, UK and aimed to systematically assess:

- What is the evidence that art therapy is clinically effective in people with non-psychotic mental health disorders?
- What is the evidence that art therapy is cost-effective in people with non-psychotic mental health disorders?

## Methods

### Search methods

Comprehensive literature searches were used to inform the clinical and cost-effectiveness reviews. A search strategy was developed to identify reviews, randomised controlled trials (RCTs), economic evaluations and all other study types relating to art therapy. Search terms were restricted to “art therapy” or “art therap$”. Methodological search filters were applied where appropriate. No other search limitations were used and all databases were searched from inception to present. Searches were conducted from May–July 2013.

Databases searched were: MEDLINE and MEDLINE in Process & Other Non-Indexed citations; EMBASE; Cochrane Library; Science Citation Index; Social Sciences Citation Index; CINAHL: Cumulative Index to Nursing and Allied Health Literature; PsycINFO; AMED: Allied and Complementary Medicine; and ASSIA: Applied Social Sciences Index and Abstracts. All resources were searched from inception to present.

### Clinical effectiveness review methods

Screening of records, study selection, and data extraction were performed by one assessor and checked by a second assessor. All studies identified for inclusion at abstract stage were obtained in full text for more detailed appraisal. Non-English studies were translated and included if relevant. Quality assessment of included studies was performed independently by two reviewers using quality assessment criteria adapted from the Cochrane risk of bias, CRD guidance, and CASP checklists to develop a modified tool to allow comprehensive and relevant quality assessment for the included trials.

The inclusion and exclusion criteria for the clinical effectiveness review are documented in Table 1.

**Table 1:** Inclusion and exclusion criteria for the systematic review

	Included	Excluded
Population	Non-psychotic clinical samples	People with psychosis
		Healthy samples
Intervention	^a^Group art therapy as might be delivered in the NHS	Other “arts therapies” including drama; music; and dance
		Play therapy
Comparator	Any including: interventions including an RCT containing art therapy; treatment as usual; waiting list; attention placebo; or other psychological therapy	None
Outcomes	Primary: treatment effectiveness; response as determined by changes in mental health rating scales;	Outcomes focussed on interpretation of the art work itself, not the participant
	Secondary: Related clinical or quality of life outcomes	
Studies	Randomised controlled trials	Non-randomised controlled studies

^a^Whilst the full health technology assessment (Uttley et al., in press) included studies of both individual and group art therapy, only studies of group art therapy are included in this paper

### Mathematical modelling methods

*A de novo* mathematical model was constructed. Due to the nature of the study question it was deemed that a complex model was not required, and that a simple model which could more clearly demonstrate the impact of key drivers of the cost effectiveness ratio would be sufficient. As such, an area under the curve model was developed to estimate the gain in utility with the following assumptions in the base case.

1. That the maximum treatment effect would be associated with the time at which treatment ended.
2. That there would be a linear increase in treatment effect, from zero at baseline to the maximum at the time at which treatment ended.
3. That there would be a residual effect of treatment with a linear decline in benefit until there was zero benefit at 52 weeks.
4. That given the short assumed duration of benefit, discounting of future costs and benefits was not necessary.

The rationale for choosing 52 weeks as the base case duration of residual benefit was based on a number of relevant references. Discussing data in Nicholson and Berman [8] and in Lambert and Ogles [9], Cooper wrote that ‘findings from the empirical research are fairly clear: clients, on average, do not tend to improve once their therapy is over...., but equally they do not tend to deteriorate rapidly’ [10]. More recent data provided in Sportel et al. [11], indicate that where Cognitive Bias Modification and Cognitive Behavioural Group Training provided larger decreases in an outcome measure (the Spielberger Test Anxiety Inventory) [12] the effect had not entirely waned at twelve month follow-up. This may be generalisable to other forms of successful psychological therapy, and conservatively we elected to assume that all benefit had dissipated at 52 weeks post treatment, although a longer period of 104 weeks was used in sensitivity analyses.

**Fig. 1:**
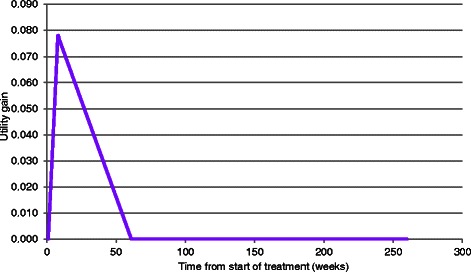
An illustration of the conceptual model of utility

The conceptual model used to calculate the utility gain across time is shown in graphical form in Fig. 1. In this figure it is assumed that there is a gain in utility of 0.0780 at week 8. The area under the curve was then translated in quality adjusted life years (QALYs) assuming 52.18 weeks per year. The QALY considers both duration and quality of life: a person living 10 years at a utility of 0.5 would accrue 5.0 undiscounted QALYs whilst a person living 8 years with a utility of 0.8 would accrue 6.4 undiscounted QALYs.

## Results and discussion

**Fig. 2:**
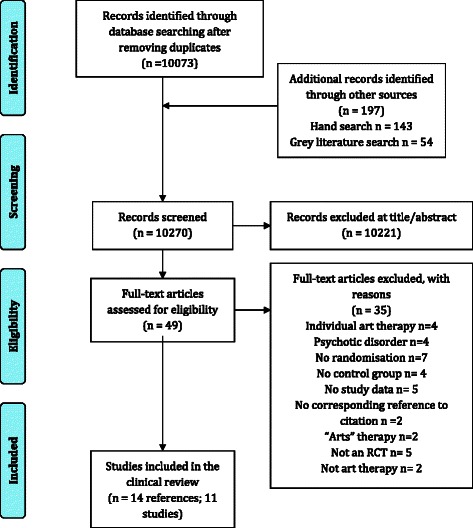
A modified PRISMA flow diagram of the studies identified and included in the clinical effectiveness review

The total number of published articles yielded from electronic database searches after duplicates were removed was 10,073. An additional 197 records were identified from supplementary searches, resulting in a total of 10,270 records for screening. Of these, 10,221 records were excluded at title/abstract screening. Figure 2 shows the flow of studies identified and included in the review.

### Clinical effectiveness results and discussion

Eleven RCTs of group art therapy were included in the clinical effectiveness review. Eight of the studies were conducted in adults and three were conducted in children. All trials had small final sample sizes with the number of participants reported to be included in each study ranging between 18 and 111. The total number of patients in the included studies is 533.

**Fig. 3:**
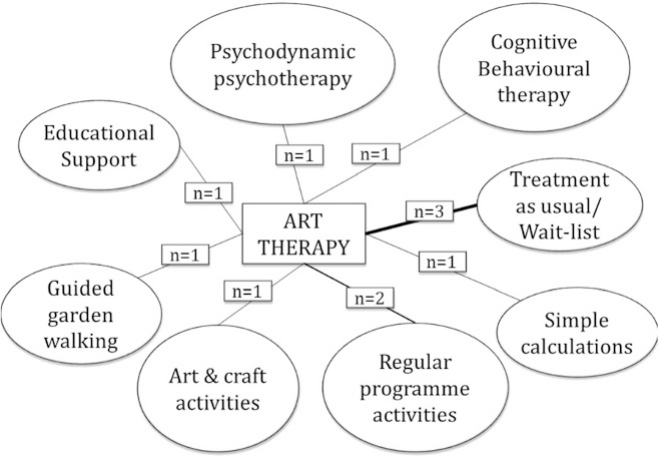
The eleven comparator arms from the included studies of group art therapy

As can be seen from Table 2 eight studies compared art therapy with an active control group. The comparator groups from the included studies can be seen in Fig. 3. Two of the studies were versus a psychological therapy (Broome [13] & Thyme [14]) whereas six studies were attention placebo control groups which mimic the amount of time and attention the intervention group receives. Three studies compared art therapy with a wait-list control or treatment as usual. The majority of studies were conducted in a community/outpatient setting, but the precise setting location for conducting the intervention was not reported in four studies (Broome [13]; Kim [15]; Monti [16]; Monti [17]) and one study was reported to be conducted in an outpatient setting (Lyshak-Stelzer et al. [18]).

**Table 2:** Characteristics of the included studies of art therapy

Study author & year	Country	Number	Patients	Art therapy description	Control description
Beebe et al. 2010 [29]	USA	22	Children with asthma	Included an opening activity; discussion of the weekly topic and art intervention; art making; opportunity for the parents to share their feelings related to the art they created, and the closing activity.	Wait-list
Broome et al. 2001 [13]	USA	97^a^	Children (*n* = 65); & adolescents (*n* = 32) with sickle cell disease	Opportunity to express feelings about pain and develop social skills through interactions with others using art as a focal point for their disease and ethnicity	Cognitive Behavioural Therapy “Relaxation” for pain or; Attention control (fun activities e.g. picnic, museum) for children group only
Gussak 2007 [30]	USA	44^a^	Incarcerated males	Asked to draw person picking an apple from a tree and other similar art therapy tasks	No treatment
Hattori et al. 2011 [24]	Japan	39	Adults with alzheimer’s disease	Primary task to colour abstract patterns which are unclear before colouring. Encouraged to draw familiar objects based on memories or favourite seasons	Simple calculations (additions and multiplications of 1 or 2 figure numbers). No pre-set target; patients completed as many as could in session
Kim 2013 [15]	Korea	50	Non-clinical older adults	Introductory 10–15 min ‘unfreezing’ phase, followed by 35–40 min for individual art making, 15–20 min for group discussion	Regular programme activities such as reading books, playing board games, and watching television
Lyshak-Stelzer et al. 2007 [18]	USA	29	Adolescents with post-traumatic stress disorder	Completion of at least 13 collages or drawings to express a “life story” narrative. Encouraged but not required to discuss dreams, memories and feelings related to their trauma	“Treatment as usual”–arts and craft making activity group
McCaffrey et al. 2011 [19]^b^	USA	39	Older adults	Drawing self-portraits; presented to group; create new drawings; display and discuss. (Art therapy was reported as the control)	The two “intervention” groups were individual (*n* = 13) or guided (*n* = 13) garden walking in the Morikami Museum and Japanese Gardens in Delray Beach
Monti et al. 2004 [31]	USA	111^a^	Women with cancer	Mindfulness based art therapy multi-modal programme including a standardised mindfulness-based stress reduction curriculum; art therapy tasks and supportive group therapy	Wait-list
Monti et al. 2006 [16]					
Monti et al. 2012 [17]	USA	18	Breast cancer (no clinical mental health problem)	Mindfulness based art therapy. Art making paired with meditation and ways of expressing emotional information in a personally meaningful manner	Educational support group: control given equal time and provided with support and resources to maximise quality of life including expert speakers on topics and time for sharing and supportive exchanges
Rusted et al. 2006 [20]	UK	45^a^	Adults with dementia	Group-interactive psychodynamic approach	Activity groups: a selection of recreational activities from different centres in the locality
Thyme et al. 2007 [14]	Sweden	39	Depressed female adults	Psychodynamic art therapy. Painting and reflective dialogue between the participant and the therapist	Verbal psychodynamic psychotherapy

^a^*N* reported is different in final sample results

^b^In this trial art therapy was designated the control arm with the two garden walking formats being designated as the interventions

The symptoms or ‘outcome domains’ under investigation and associated outcome measures are reported in Table 3.

**Table 3:** Outcomes investigated in the eleven included studies

Study author & year	Outcome domains investigated	Outcome measures	Time points
Beebe 2010 [29]	Quality of life; behavioural & emotional adaptation	Paediatric Quality of Life (PedsQL) Asthma module Beck Youth Inventories–Second Edition	7 weeks & 6 months
Broome 2001 [13]	Coping and health care utilisation	Schoolagers Coping Strategies Inventory (SCSI) Adolescent Coping Orientation for Problem Experiences (A-COPE) ER visits; clinic visits; hospital admissions	4 weeks & 12 months
Gussak 2007 [30]	Depression	Beck Depression Inventory-Short Form (BDI)	Exact time point NR “Post test”
Hattori 2011 [24]	Mood; vitality; behavioural impairment; QoL; activities of daily living (ADL); cognitive function	Mini-Mental State Examination Score (MMSE) Wechler Memory Scale revised (WMS-R) Geriatric Depression Scale (GDS); Apathy Scale (Japanese version) SF-8–Physical (PCS-8)& Mental (MCS-8) components Barthel Index Dementia Behaviour Disturbance Scale (DBD) Zarit Caregiver Burden Interview	12 weeks
Kim 2013 [15]	Positive/negative affect; state-trait anxiety; self-esteem	Positive & Negative Affect Schedule (PANAS) State-Trait Anxiety Inventory (STAI) Rosenberg Self = Esteem Scale (RSES)	NR: assume 4 weeks
Lyshak-Stelzer 2007 [18]	PTSD symptoms	UCLA PTSD Reaction Index for DSM-IV Child Version Milieu behavioural measures e.g. use of restraints	NR: reports (*n*)s for 2 years. Study is ongoing in a further 15 patients
McCaffrey et al. 2011 [19]	Depression	Geriatric Depression Scale (GDS)	6 weeks
Monti 2006 [16]	Symptoms of distress including depression, anxiety and quality of life	Symptom Checklist 90 Revised (SCL-90-R) Global Severity Index (GSI) Medical Outcomes Study Short-Form Health Survey (SF-36)	8 & 16 weeks
Monti 2012 [17]	Correlation of CBF on fMRI with experimental condition	fMRI Cerebral blood flow (CBF) and correlation with anxiety using Symptom Checklist-90-Revised (SCL-90-R)	Within 2 weeks of end of 8-week programme
Rusted 2006 [20]	Depression; mood; sociability and physical involvement	Cornell Scale for Depression in Dementia (CSDD) The Multi Observational Scale for the Elderly (MOSES) The Mini-Mental State Exam (MMSE) The Rivermead Behavioural Memory Test (RBMIT) Tests of Everyday Attention (TEA) Benton Fluency Task	10; 20 & 40 weeks during trial then 44 & 56 week follow up
Thyme 2007 [14]	Stress reactions after a range of traumatic events; Mental health symptoms; Depression	Impact of Event Scale (IES) Symptom Check List (SCL-90) Beck Depression Inventory (BDI) Hamilton Rating Scale of Depression (HRSD)	10 weeks & 3 month follow up

The study populations were heterogeneous in their clinical profiles highlighting the wide application of art therapy but also demonstrating the difficulty in obtaining a pooled estimate of treatment effect. The control groups across the included studies are heterogeneous therefore there may be different estimates of treatment effects depending on what art therapy is compared against. Additionally, despite common mental health symptoms being investigated across the included RCTs, the majority of studies were using different measurement scales to assess these outcomes (see Table 3). Therefore as there is insufficient comparable data on outcome measure across studies it is not possible to perform a formal pooled analysis.

Potential treatment effect modifiers include the experience/qualification of the art therapist, characteristics that were not consistently reported. Also, the age of the included patients could be a potential effect modifier as eight studies are of adults and three are of children. Pre-existing physical conditions were present in seven of the included studies which could also represent a potential treatment effect modifier.

The direction of statistically significant results from the 15 included RCTs are summarised in Table 4.

**Table 4:** Summary of the direction of findings from the eleven included studies

Direction of significant findings	Number	Studies
Significant positive effects in all outcome measurements investigated in the art therapy group compared to the control group	1	Kim 2013 [15]
Significant positive effects in some, but not all, outcome measurements investigated in the art therapy group compared to the control group.	6	Beebe 2010 [29]
		Gussak 2007 [30]
		Hattori 2011^a^ [24]
		Lyshak-Stelzer 2007 [18]
		Monti 2006 [16]
		Monti 2012 [17]
Improvement from baseline but no significant difference between groups	3	Broome 2001 [13]
		McCaffrey 2011 [19]
		Thyme 2007 [14]
Art therapy worse than comparator at baseline and follow-up	1	Rusted 2006 [20]

^a^Reported a significant positive effect for control group on one outcome measurement

As can be seen in Table 4, in 10 of the 11 included studies there were improvements from baseline in some outcomes in the art therapy groups. However, both the intervention and the control groups improved from baseline in three studies with no significant difference between the groups (Broome [13]; McCaffrey [19] and Thyme [14]). The control groups across these three studies were: CBT; garden walking; and verbal psychodynamic psychotherapy respectively. In six studies art therapy was significantly better than the control group for some but not all outcome measures. Table 5 shows the results according to the mean change from baseline between groups in these six studies.

**Table 5:** Six included studies with statistically significant findings in the art therapy group in some but not all outcome measures

Study & control description	Outcome measures: mean changes from baseline (CFB) and *p* values
Beebe 2010 [29]	Paediatric Quality of Life (PedsQL) Asthma module
Wait list	Intervention positive reduction in 4/10 QoL items at 7 weeks:
	Between groups means at 7 weeks
	QoL–Parent total (6.167 vs −13.091) *p* = 0.025; QoL–Child total (9.727 vs −13.364) *p* = 0.0123; QoL–Parent worry (47.917 vs −13.182) *p* = 0.0144; QoL–Child worry (54.545 vs −45.909) *p* = 0.0142
	Intervention positive reduction in 2/10 at 6 months:
	Between groups means at 6 months:
	QoL–Parent worry (58.333 vs −40.909) *p* = 0.024; QoL–Child worry (79.545 vs −25.000) *p* = 0.0279
	Beck Youth Inventories–Second Edition
	Intervention significant reduction in 2/5 items at 7 weeks compared to control:
	Beck–Anxiety (−15.6 vs 5.3) *p* = 0.0388; Beck–Self-concept (12.091 vs −3.545) *p* = 0.0222
	Intervention significant reduction 1/5 at 6 months:
	Beck–Anxiety (−14 vs 0.545) *p* = 0.03
	No significant differences for depression component of Beck youth inventory at 7 weeks (*p* = 0.21) or 6 months (*p* = 0.29)
	Baseline means NR
Gussak 2007 [30]	Beck Depression Inventory-Short Form (BDI)
Treatment as usual	Statistically significantly greater decrease in intervention compared to control:
	BDI Intervention mean CFB (−7.81) vs Control (+1.0) *p* < 0.05
Hattori 2011 [24]	SF-8–Physical (PCS-8) & Mental (MCS-8)
Simple calculations	Intervention significant improvement from baseline in MCS-8 subscale of SF-8 components:
	Percentage of patients showing a 10 % > improvement was compared between groups by chi-squared test. MCS-8 (*p* = 0.038; odds ratio, 5.54)
	Apathy Scale (Japanese version)
	Statistically significant improvement from baseline (*p* = 0.0014) in Apathy scale but not significantly different to control:
	CFB Intervention (−3.2) vs Control (−1.1) *p* = 0.09
	Mini-Mental State Examination Score (MMSE)
	Control group significant improvement in MMSE compared to art therapy intervention:
	CFB Intervention (−0.02) vs Control (+1.1) *p* < 0.01
	Wechler Memory Scale revised (WMS-R); Geriatric Depression Scale (GDS) Barthel Index; Dementia Behaviour Disturbance Scale (DBD); Zarit Caregiver Burden Interview
	No significant differences in other items
Lyshak-Stelzer 2007 [18]	UCLA PTSD Reaction Index for DSM-IV Child Version
Arts and craft	Intervention significantly better at reducing trauma symptoms than Control:
	CFB Int (−20.8) vs Con (−2.5) *p* < 0.01
	Milieu behavioural measures e.g. use of restraints
	No significant differences for behavioural milieu
Monti 2006 [16]	Global Severity Index (GSI)
Wait-list	Intervention had significantly decreased symptoms of distress and highly significant improvements in some QoL areas: compared to control: GSI CFB Int (−0.20) vs Con (−0.04) *p* < 0.001
	Symptom Checklist 90 Revised (SCL-90-R)
	SCL-90-R CFB: Anxiety Int (−0.26) vs Con (−0.10) *p* = 0.02; Depression Int (−0.27) vs Con (−0.08) *p* = 0.01
	Medical Outcomes Study Short-Form Health Survey (SF-36)
	SF36: General health Int (7.97) vs Con (−.59) *p* = 0.008; Mental health Int (13.05) vs Con (2.16) *p* < 0.001
Monti 2012 [17]	Symptom Checklist-90-Revised (SCL-90-R)
Educational support group	Anxiety reduced in Int but not control group:
	SCL-90-R decrease in Int (*p* = 0.03) but not in Con (*p* = 0.09)
	fMRI Cerebral blood flow (CBF) and correlation with anxiety using CBF
	fMRI changed in certain brain areas in art therapy group only.
	No changes in control group

In one study (Kim [15]) outcomes for the art therapy intervention group were significantly better than the control group for all outcomes. Table 6 shows the results from the Kim 2013 study [15].

**Table 6:** One included study with statistically positive findings for all outcomes in the art therapy group

Study & control description	Outcome measures and results
Kim 2013 [15]	Significant improvements for Intervention in all three outcomes compared to Control
Regular programme activities	Positive & Negative Affect Schedule (PANAS)
	PANAS CFB Intervention (19.88) vs Control (−5.64) *p* < 0.01
	State-Trait Anxiety Inventory (STAI)
	CFB STAI State (−13.17) vs (+3.08) *p* < 0.01
	CFB STAI Trait (−7.84) vs (+2.96) *p* < 0.01
	Rosenberg Self = Esteem Scale (RSES)
	CFB RSES (4.24) vs (−0.48) *p* < 0.01

In one study (Rusted [20]) from a sample of people with dementia, outcomes were worse for the art therapy group than the control group, which was an activity control group. An unusual pattern of results is presented including a significant increase reported in anxious/depressed mood (*p* < 0.01) at 40 weeks which is not present at the 10 or 20 week time points and dissipates by 44 and 56 weeks. The authors discuss several reasons for this result including the high level of attrition; the reliance on observer ratings in the frail and elderly sample (and subsequent potential impact of observer bias); the increased depression as a response to the sessions ending; and the possibility that this sample were contra-indicated for art therapy.

Adverse events were not reported in any of the included RCTs. The lack of adverse event data in the majority of included studies is not necessarily evidence that there were no adverse events in the included trials, it may only be an indication that adverse events were not recorded. Potential harms and negative effects of art therapy are further explored in the qualitative review within the full health technology assessment (Uttley et al. (in press)).

Quality assessment of the 11 included RCTs indicated that the trials were generally of low quality (see Table 7). All trials had high or unclear risk of bias across several domains particularly for: method of randomisation; allocation concealment; blinding; detection bias; and incomplete outcome data.

**Table 7:** Summary of risk of bias (high, low or unclear) in the 11 included quantitative studies

Study	Sequence generation	Treatment allocation concealment	Performance bias (participant blind)	Detection bias (outcome assessment blind)	Baseline comparability	Groups treated equally	Selective outcome reporting	Incomplete outcome data	Researcher allegiance
Beebe 2010 [29]	Unclear	Unclear	High	Low	Unclear	High	Low	Unclear	Low
Broome 2001 [13]	Unclear	Unclear	High	Unclear	Unclear	Low	Low	Unclear	Low
Gussak 2007 [30]	Unclear	Unclear	High	Unclear	High	High	Low	Unclear	High
Hattori 2011 [24]	Unclear	Unclear	High	Unclear	Low	Low	Low	Low	Low
Kim 2013 [15]	Unclear	Unclear	High	High	Low	Low	Low	Low	High
Lyshak-Stelzer 2007 [18]	Unclear	Unclear	High	Unclear	Low	Low	Low	High	Low
McCaffrey 2011 [19]	Low	Unclear	High	Unclear	Low	Low	Low	Low	High
Monti 2006 [16]	Unclear	Unclear	High	Unclear	High	High	Low	Low	Low
Monti 2012 [17]	Unclear	Unclear	High	Unclear	Low	Low	Low	High	Low
Rusted 2006 [20]	Unclear	Unclear	High	Unclear	High	Low	Low	High	Low
Thyme 2007 [14]	Low	Low	High	Unclear	Unclear	Low	Low	Low	Low

In addition, withdrawals were not consistently reported or accounted for in the included trials which are particularly important considering the small sample sizes in the included trials. Therefore attrition in the studies represents an important confounder. Also concomitant treatment and treatment fidelity which were rarely reported, represent additional possible confounders to the review findings.

### Cost-effectiveness results and discussion

During the clinical effectiveness review 192 abstracts were identified that were potentially relevant for cost-effectiveness purposes and these were reviewed by a health economic modeller. Twenty six articles were retrieved for detailed inspection, although only 1 was deemed relevant (12 were not art therapy; 9 contained no economic data; 4 non-English text).

No existing models of art therapy were identified. One paper that was deemed as potentially relevant was not an economic appraisal but did report costs incurred and health related benefits pertaining to a single patient over a 6 year period [21]. This patient was one of 357 patients initially recruited but the paper did not discuss the potential impact of selection bias on the results presented.

To follow recommended National Institute of Health and Care Excellence (NICE) guidelines [22] for conducting economic evaluations the health of patients should use a preference based utility measure. Utility is a measure of patient health where 0 equates to death and 1 equates to perfect health. The Euroqol 5 dimensions (EQ-5D) is the preferred measure by NICE. None of the RCTs identified included a preference-based utility measure and therefore mappings from outcome measures reported in the RCTs to the EQ-5D were sought from an online database (http://www.hqlo.com/content/11/1/151) reported by Dakin [23]. Two outcome measures in the RCTs could be mapped onto the EQ-5D: the medical outcomes short form (36) health survey (SF-36) reported in Monti et al. [16] and the Barthel Index reported in Hattori et al. [24]. However, in Hattori et al. [24] the Barthel index is reported for the overall score only whereas mapping to the EQ-5D would require the individual component scores. The authors were contacted to enquire whether the individual component data could be obtained, however, the authors declined to provide these data due to their intention to publish these in a forthcoming publication.

In the Monti et al. RCT all participants had a diagnosis of breast cancer, with participants between 4 months and 2 years post-diagnosis. Women with a terminal diagnosis, or who had a current diagnosis of a major mood disorder, psychotic disorder or significant cognitive deficit were excluded. Those receiving any type of mental health care could be included but had to obtain written permission from their treating health professional to enter the study. Eight week data from Monti et al. were available and the SF-36 data reported are shown in Table 8. Only those variables that have been used in the mapping algorithms have been reported.

**Table 8:** The SF-36 data as reported in Monti et al. [16]

SF-36 scale	Wait-list control arm (*n* = 55)		Art therapy arm (*n* = 56)		Change over the 8-week period		Difference in change (Art therapy–wait-list control) over an 8 week period (95 % CI)
	Week 0	Week 8	Week 0	Week 8	Wait-list control	Art therapy	
Physical functioning	64.37	64.42	58.23	65.01	0.05	6.78	6.73 (−13.8, 0.37)
Social functioning	60.04	64.91	51.22	66.60	4.87	15.38	10.51 (−20.9, −0.10)
Physical role^a^	0.00	25.00	0.00	50.00	25.00	50.00	0.00
Emotional role	33.33	66.67	33.33	66.67	33.34	33.34	0.00
Mental health	64.91	67.07	56.90	69.95	2.16	13.05	10.89 (−16.8, −4.96)
Vitality	42.63	42.91	40.26	50.06	0.28	9.80	9.52 (−16.7, −2.37)
Bodily pain	58.14	58.74	54.23	60.14	0.60	5.91	5.31 (−14.1, 3.50)
General health	55.78	55.19	47.13	55.09	−0.59	7.96	8.56 (−14.8, −2.29)

^a^For discussion of the inconsistency in this scale see main text

Two mapping algorithms from SF-36 to EQ-5D were identified: one by Ara and Brazier [25] and one by Rowen et al. [26], these predicted utility gains at the end of the 8-week period of 0.0780 and 0.0871 respectively using the data in Table 8. As the Monti et al. RCT also reported changes in the Global Severity Index (GSI) [27], the summary score from the Symptoms Checklist Revised measure, an inference could be made between a unit decrease in GSI and utility gain estimated via mapping: this value was 0.487 using the Ara and Brazier [25] mapping and 0.542 using the Rowen et al. [26] mapping. As GSI data were presented in Thyme et al. [14] this trial could now be used in an economic evaluation, albeit with more uncertainty in the generated results. It was estimated that at the end of the 10-week treatment period in Thyme et al. [14] there was a utility loss associated with short-term psychodynamic art therapy compared with short-term psychodynamic verbal therapy, henceforth, abbreviated to verbal therapy. This value was 0.122 using the Ara and Brazier [25] algorithm and 0125 using the Rowen et al. [26] algorithm.

Attempts were made to make further inferences on utility changes from the changes in the remaining outcome measures reported in the Thyme et al. [14] RCT in order to widen the number of RCTs considered but this did not allow the inclusion of further RCTs in the economic evaluation.

Due to heterogeneity the two RCTs were analysed separately. Based on clinical advice regarding the generalisability of the RCTs to practice in England and Wales and limitations of the Thyme et al. [14] RCT (see later) the results from the Monti et al. [16] RCT was set to be the primary analyses with results from Thyme et al. [14] denoted exploratory analyses.

Within the Monti et al. RCT the costs of art therapy per woman was assumed to be £180 using data from the British Association of Art Therapists (BAAT) (personal communication Val Huet, British Association of Art Therapists, February 2014) and £248 using data reported by Curtis [28]. For Thyme et al. the cost per participant was £80 (BAAT) and £110 (Curtis [28]). The cost of the verbal therapy in Thyme et al. [14] was estimated to be £64 (BAAT) and £88 (Curtis [28]) per participant, assuming a verbal therapist had the same cost as an art therapist. It was assumed that control/waitlist incurred no cost in therapist time. Full details on the methods for estimating costs are provided in Uttley et al. (in press).

Probabilistic sensitivity analyses were undertaken to generate the expected cost per QALY for each RCT using the distributions reported in Table 9. It was assumed that all distributions were independent. Scenario analyses were undertaken using: the Ara and Brazier [25] and Rowen et al. [26] mapping algorithms; the BAAT and Curtis [28] cost estimations; and 52 and 104 week residual benefits.

**Table 9:** The distributions used in the probabilistic sensitivity analyses

	Mean value	2.5^th^ percentile	97.5^th^ percentile
Utility gain in the Monti et al. [16], RCT of art therapy compared with wait-list control^a^	0.078	0.034	0.119
Utility gain in the Monti et al. [17], RCT of art therapy compared with wait-list control^b^	0.087	0.043	0.126
Relationship between one unit decrease in GSI and utility gain (using Ara and Brazier [25])	0.485	0.212	0.744
Relationship between one unit decrease in GSI and utility gain (using Rowen et al. [26])	0.542	0.271	0.790
GSI decrease of verbal therapy compared with art therapy in the Thyme et al. [14], RCT.	0.235	−0.270	0.721
	(Verbal therapy more effective)	(Art therapy more effective)	(Verbal therapy more effective)
Derived utility gain in the Thyme et al. [14], RCT of verbal therapy compared with art therapy (using Ara and Brazier [25])	0.114	−0.145	0.386
Derived utility gain in the Thyme et al. [14], RCT of verbal therapy compared with art therapy (using Rowen et al. [26])	0.127	−0.160	0.426

^a^Having sampled from the SF-36 dimensions and mapped to utility using the Ara and Brazier [25] algorithm

^b^Having sampled from the SF-36 dimensions and mapped to utility using the Rowen et al. [26], algorithm

In addition, a threshold analysis was conducted to ascertain the likely level of gain in utility at 52 weeks that would be required for art therapy, as typically used in England and Wales, to be deemed cost effective compared with wait list. This used £20,000 per QALY gained, which is a threshold cited by NICE [22] as signifying an intervention is likely to be cost effective. To undertake this analysis assumptions regarding the likely cost, and likely durations of treatment and residual benefit were required. Whilst it is acknowledged that there is a spectrum of needs and treatments it was believed that the majority of patients would be treated in either an art therapy outpatient group or a community recovery setting, with only a small proportion needing more expensive treatment. Using data provided from the BAAT, it was assumed that typical treatment would be of 42 sessions, over a 52-week period and with a cost, per patient, of £750.

#### Primary results from the model

##### Monti et al. (2006) [16]

**Fig. 4:**
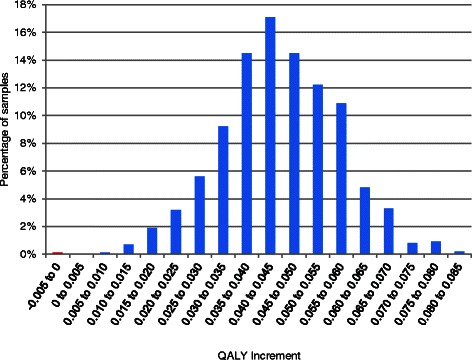
A histogram of the utility gain of art therapy compared with wait list estimated from the Monti et al. [16], RCT, mapping from Ara and Brazier [25] and assuming 52 weeks’ residual benefit and costs per patient from Curtis [28]

Probabilistic results for the Monti et al. RCT are shown in Table 10. It is seen that even in unfavourable scenarios (low residual benefit and increased cost per participant and using the Ara and Brazier [25] algorithm) the expected cost per QALY is below £6000. A histogram of the QALY benefit associated with art therapy is shown in Fig. 4.

**Table 10:** Probabilistic results from the Monti et al. [16] scenario: art therapy versus wait list

		Using the Ara and Brazier (2008) [25] mapping			Using the Rowen et al. (2009) [26] mapping		
Duration of residual benefit	Costing source	Inc costs (£)	Inc QALY	Cost per QALY (£) (95 % CI)	Inc costs (£)	Inc QALY	Cost per QALY (£) (95 % CI)
52 weeks	BAAT	180	0.0447	4031 (2628–9202)	180	0.0499	3610 (2477–7229)
52 weeks	Curtis [28]	248	0.0447	5542 (3613–12,653)	248	0.0499	4963 (3405–9940)
104 weeks	BAAT	180	0.0834	2159 (1408–4930)	180	0.0931	1934 (1327–3873)
104 weeks	Curtis [28]	248	0.0834	2969 (1936–6779)	248	0.0931	2659 (1824–5325)

*Inc* Incremental

##### Threshold analysis

In the threshold analysis it was calculated that even with unfavourable assumptions regarding length of residual benefit and mapping algorithm the utility gain required to be cost effective would be below 0.04. This value is below that reported by Monti et al. [16], which had a mean value of 0.078 indicating that art therapy as practiced in England and Wales was likely to be seen as cost effective compared with wait list.

#### Exploratory results from the model

##### Thyme et al. (2007) [14]

**Fig. 5:**
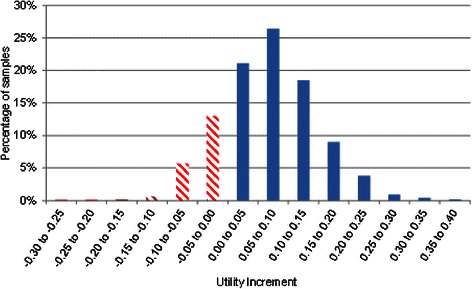
A histogram of the utility gain of verbal therapy compared with art therapy estimated from the Thyme et al. [14], RCT, mapping from Ara and Brazier [25] and assuming 52 weeks’ residual benefit and costs per patient from Curtis [28]

Probabilistic results when using data from the Thyme et al. [14] RCT are shown in Table 11. It is seen that the expectation is that verbal therapy dominates art therapy as it is marginally cheaper and more efficacious. However, there is considerable uncertainty and the 95 % confidence intervals indicate that art therapy may have a cost per QALY gained compared with verbal therapy of less than £300. A histogram of the incremental benefit of verbal therapy compared with art therapy is shown in Fig. 5: this shows considerable uncertainty in the most effective intervention with the solid blue bars indicating verbal therapy is more cost effective and the striped red bars indicating that art therapy is more cost effective. Art therapy is the more efficacious intervention in approximately 20 % of simulations.

**Table 11:** Probabilistic results from the Thyme et al. [14] scenario: verbal therapy versus art therapy

		Using the Ara and Brazier (2008) mapping [25]			Using the Rowen et al. (2009) mapping [26]		
Duration of residual benefit	Costing Source	Inc costs (£)	Inc QALY	Cost per QALY (£) (95 % CI)	Inc costs (£)	Inc QALY	Cost per QALY (£) (95 % CI)
52 weeks	BAAT	−16	0.0675	Dominating (Dominating–183^a^)	−16	0.0757	Dominating (Dominating–99^a^)
52 weeks	Curtis [28]	−22	0.0675	Dominating (Dominating–251^a^)	−22	0.0757	Dominating (Dominating–136^a^)
104 weeks	BAAT	−16	0.1241	Dominating (Dominating–168^a^)	−16	0.1391	Dominating (Dominating–91^a^)
104 weeks	Curtis [28]	−22	0.1241	Dominating (Dominating–230^a^)	−22	0.1391	Dominating (Dominating–125^a^)

N.B.: Dominated means both more expensive and less efficacious

*Inc* Incremental

^a^These values represent cost per QALY lost and as such these upper bound values indicate art therapy is more cost effective than verbal therapy

Evidence from two RCTs has been used to generate estimates of cost effectiveness, although there are caveats regarding: the mappings; the study population; small sample sizes; and possible confounding, all of which increase the uncertainty in our results.

The Monti et al. [16] RCT recruited women with breast cancer, of varying stages, and with a range of time since diagnosis between 4 months and 2 years and was conducted in the USA. The generalisability of these women to those treated with art therapy in England and Wales is unclear. Furthermore, there may be inaccuracy introduced by the values in Table 8. It is noted that the data for physical role and emotional roles at week 8, are medians (and change in the median) due to the non-normality of the data whereas means would be preferable. There also is a discrepancy in the results for the physical role scale, as the values reported at weeks 0 and 8 weeks indicate a change of 25 across the 8 week period (50–25) yet the reported difference was zero. We assumed that the value of zero reported for the change between art therapy and wait list is correct, which could be unfavourable to art therapy. A further caveat regarding the reliability of these efficacy data is that only women with values at baseline (week 0) and at end of treatment (week 8) were included in the analysis with no imputation for missing data. There were 11 dropouts in the art therapy arm and 7 dropouts in the control arm. If these reported dropouts were not random but related to lack of (perceived) efficacy then it is possible that the reported results favour art therapy.

The Thyme et al. [14] study compared art therapy and verbal therapy. The RCT was conducted in Sweden and recruited 44 women. At recruitment, 28 (63.6 %) study participants were diagnosed with dysthymic disorder and 16 (36.4 %) study participants had depressive symptoms and difficulties. One participant withdrew her participation before randomisation resulting in a final study population at randomisation of 43 women, (21 art therapy; 22 verbal therapy). Of these, 39 women completed the study (*n* = 18 art therapy; *n* = 21 verbal therapy). The reported results are potentially confounded by concomitant treatment; two participants in the verbal therapy intervention “accepted body awareness as an additional treatment during psychotherapy” compared with none in the art therapy arm. The mechanism by which these women were offered body awareness is unclear. In addition, the use of anti-depressants may differ between arms as the text is unclear: “In the AT group, one participant were (sic) prescribed antidepressants during therapy (*n* = 1) and one between termination of therapy and the 3-month follow-up (*n* = 1), and in the VT group three during therapy (*n* = 1) and two after (*n* = 2).” Data from women who dropped out from the study (*n* = 2 art therapy; *n* = 1 verbal therapy) or who were referred for long-term art psychotherapy (*n* = 1 art therapy; *n* = 0 verbal therapy) were not included in the analysis which may add uncertainty to the results. It is noted that as two active interventions were trialled no inference could be made with respect to the relative efficacy compared with no treatment.

### Limitations of the work

This review can be considered as an evidence portfolio for art therapy across several non-psychotic mental health disorders but as such it suffers from substantial heterogeneity in the patient clinical profiles included. Focusing the population of interest to specific health conditions or outcome domains in future systematic reviews will increase the precision of any resulting pooled treatment effects.

## Conclusions

From the limited number of studies identified in patients with different clinical profiles, art therapy was reported to have statistically significant positive effects compared with control in a number of studies. It was not possible to produce pooled estimates of the clinical effectiveness of group art therapy due to heterogeneity in the data. The risk assessment of bias highlighted that the quality of the included trials was generally low and prone to areas of potential confounding. Subsequently the internal validity of the included studies is threatened. The results from the clinical effectiveness review should be interpreted with caution due to the generally low quality of the small number of RCTs identified and the small sample sizes involved.

It appears that art therapy is likely to be cost effective compared to wait list. Using expected values art therapy appears to be dominated by verbal therapy although there is considerable uncertainty, with art therapy being the more cost effective treatment in approximately 20 % of simulations. Given this, and the limitations of the evidence for art therapy versus verbal therapy RCT, no definitive statement can be made regarding this comparison.

## Abbreviations

CRD: Centre for Reviews and Dissemination–University of York
CASP: Critical Appraisal Skills Programme
CI: Confidence interval
EQ-5D: EuroQol five-dimension health instrument
GSI: Global severity index
NHS: National Health Service
NICE: National Institute for Health and Care Excellence
NR: Not reported
PRISMA: Preferred reporting items for systematic reviews and meta-analyses
PSA: Probabilistic sensitivity analysis
QALY: Quality-adjusted life-year
QoL: Quality of life
RCT: Randomised controlled trial
SCL-90-R: Symptoms checklist revised
SF-36: Medical outcomes short form (36) health survey
UK: United Kingdom
