# Erratum to: The clinical and cost effectiveness of group art therapy for people with non-psychotic mental health disorders: a systematic review and cost-effectiveness analysis

**DOI:** 10.1186/s12888-015-0599-2

**Published:** 2015-09-10

**Authors:** Lesley Uttley, Matt Stevenson, Alison Scope, Andrew Rawdin, Anthea Sutton

**Affiliations:** School of Health and Related Research (ScHARR), University of Sheffield, Regent Court, 30 Regent Street, Sheffield, S1 4DA UK

Unfortunately, the original version of this article [1] contained an error. An entry was incorrect *Uttley et al.* in the reference list. The correct reference can be found below:

Uttley L, Scope A, Stevenson M, Rawdin A, Taylor Buck E, Sutton A, et al. Systematic review and economic modelling of the clinical effectiveness and cost-effectiveness of art therapy among people with non-psychotic mental health disorders. Health Technol Assess 2015;19(18).
